# Polymeric Wet-Strength Agents in the Paper Industry: An Overview of Mechanisms and Current Challenges

**DOI:** 10.3390/ijms24119268

**Published:** 2023-05-25

**Authors:** Iolanda Francolini, Luciano Galantini, Fernando Rea, Cristiano Di Cosimo, Pierpaolo Di Cosimo

**Affiliations:** 1 Department of Chemistry, Sapienza University of Rome, Piazzale A. Moro, 00185 Rome, Italy; luciano.galantini@uniroma1.it; 2 Gima Water & Air s.R.l (GWA), Via Fratta Rotonda Vado Largo, 03012 Anagni, Italy; f.rea@g-wa.it (F.R.); c.dicosimo@g-wa.it (C.D.C.); p.dicosimo@g-wa.it (P.D.C.)

**Keywords:** paper packaging, polyamideamine-epichlorydrin resin, chitosan, bio-based wet-strength agents

## Abstract

Polymeric wet-strength agents are important additives used in the paper industry to improve the mechanical properties of paper products, especially when they come into contact with water. These agents play a crucial role in enhancing the durability, strength, and dimensional stability of paper products. The aim of this review is to provide an overview of the different types of wet-strength agents available and their mechanisms of action. We will also discuss the challenges associated with the use of wet-strength agents and the recent advances in the development of more sustainable and environmentally friendly agents. As the demand for more sustainable and durable paper products continues to grow, the use of wet-strength agents is expected to increase in the coming years.

## 1. Introduction

For centuries, the paper industry has been a crucial player in our daily lives, providing us with essential materials such as printing and writing paper, tissue paper, and packaging products. With a global market size of over $500 billion, the paper industry holds a significant position globally and employs millions of people worldwide, contributing significantly to the economies of many countries. The paper manufacturing industries employ around 647,000 workers in 21,000 companies. In the EU, the annual turnover from the production of pulp, as well as graphic and hygienic packaging and specialized paper grades and products is around EUR 180 billion [1]. The global pulp and paper market is projected to grow from USD 354.39 billion in 2022 to USD 372.70 billion by 2029, exhibiting a growth rate of 0.72% over the period forecast 2022–2029 [2].

Europe is a significant producer of paper. Roughly 50% of paper production is dedicated to packaging, while the remaining half is evenly split between the production of paper for graphic applications and paper for sanitary products [3].

Despite challenges related to sustainability and changing consumer demands, the paper industry continues to thrive and evolve [4]. Paper is a highly versatile material used for packaging due to its light weight, easy handling, and flexibility. It is also environmentally friendly, being sustainable and recyclable. In this context, in recent years, there has been a growing demand for sustainable packaging solutions, and paper has emerged as a popular choice [5]. Paper-based packaging has several advantages over traditional plastic packaging as it is biodegradable, made from a renewable resource, and easy to recycle [6]. With the growing demand for sustainable packaging solutions, it is likely that we will see even more innovative uses of paper in packaging in the years to come.

**Figure 1:**
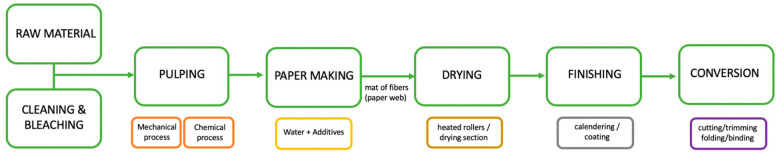
Simplified scheme of the process of paper making. The additives, including the wet-strength agents, are added to the pulp in the paper making step, before the formation of the mat of fibers.

The process of making paper starts with raw materials such as wood, recycled paper, and agricultural fibers, which are processed into pulp through mechanical or chemical methods [7,8]. The pulp is then mixed with water to form a slurry, which is spread out on a screen, drained of water, and left to dry, press, and finish the paper. The finished product can then be cut into sheets or rolled into large reels for various uses (Figure 1). The demand for high-quality paper products continues to grow, and paper manufacturers are constantly striving to improve the properties of their products.

One of the key properties that is highly valued in paper products is their wet strength, which refers to the ability of the paper to maintain its strength and integrity when exposed to moisture [9]. To achieve this, wet-strength agents are added to the paper during the manufacturing process. Most of the common wet-strength agents are added to the pulp before the sheet is formed at the “wet end” of the machine (wet-end addition). Additives that are not absorbed by paper fibers must be added to the paper after sheet formation (Figure 1).

Polymer wet-strength agents are of outmost importance in the paper industry due to their ability to enhance water resistance, dimensional stability, durability, and cost-effectiveness of paper products. These agents are chemical additives working by forming cross-linking bonds between the paper fibers, creating a network that reinforces the paper structure [10]. This results in enhanced water resistance, preventing the paper from becoming weak or disintegrating when exposed to liquids. This property is particularly crucial for various paper-based applications, such as packaging materials, labels, and tissues, where exposure to moisture is common. It also enables the use of cellulose in membrane technology for removing contaminants from water. Current membrane technology involves the use of expensive synthetic materials. In contrast, emerging cellulose membrane technology can provide a low-cost platform for various pressure-driven filtration techniques, such as microfiltration, ultrafiltration, and reverse osmosis [11].

In addition to water resistance, polymer wet-strength agents contribute significantly to the dimensional stability of paper products. These agents strengthen the inter-fiber bonds, making the paper more resistant to dimensional changes, not only when exposed to varying levels of humidity or moisture, but also in dry conditions [12]. A nearly linear improvement in both the wet and dry strength of softwood bleached Kraft pulp can be achieved by increasing the dosage of polymer wet-strength agents up to 1%. However, beyond this dosage, the increase in wet strength becomes marginal, while dry strength remains largely unaffected [12]. As a result, the paper retains its original shape, size, and flatness, ensuring that it remains visually appealing and functional. Dimensional stability is particularly important for applications such as printing, converting, and packaging, where precise and accurate dimensions are required.

The increase in strength and resilience provided by these agents results in paper that is more resistant to tearing, breaking, or puncturing, thus enhancing the durability and longevity of paper products. They improve the tensile strength and tear resistance of the paper fibers, making the paper more robust and able to withstand rigorous handling, printing, and converting processes. Additionally, polymer wet-strength agents help to maintain the integrity of the paper when it is exposed to repeated wetting and drying cycles. This is particularly important for applications such as paper towels, tissues, and wipes.

Finally, the use of polymer wet-strength agents offers advantages in terms of production efficiency and cost savings. During the paper manufacturing process, excessive drying is often required to remove moisture from the paper, which consumes significant amounts of energy and increases production costs. However, the application of wet-strength agents can reduce the need for excessive drying by improving the paper resistance to moisture. This not only saves energy but also improves production efficiency by reducing drying times and increasing throughput. Additionally, by minimizing the risk of paper damage during wetting and drying processes, polymer wet-strength agents contribute to a decrease in waste and rejections, resulting in cost savings for paper manufacturers.

There are various wet-strength agents available on the market, either synthetic or natural, each with its own specific mechanism of action and benefits. In this review, we aim to provide an overview of the different types of wet-strength agents, including their chemical structures and mechanisms of action. We will also discuss the challenges associated with the use of wet-strength agents, such as their potential impact on the environment and their role in the transition towards a sustainable and circular economy.

## 2. Types of Wet-Strength Agents

Wet-strength agents are polymers that are added to paper products to improve their strength and dimensional stability in the presence of moisture. These agents play a vital role in the paper industry and are typically added to paper products such as tissue paper, paper towels, and filter paper, which are likely to come in contact with water during use.

There are two main types of wet-strength agents used in the paper industry: synthetic and natural (Table 1).

Synthetic wet-strength agents are typically based on polymers and include polyamideamine epichlorohydrin, polyacrylamide, and polyethyleneimine. These agents work by crosslinking with cellulose fibers in the paper, forming a strong network that resists the breakdown of the paper when it comes in contact with water.

Natural wet-strength agents, on the other hand, are derived from natural sources such as starch and chitosan. These agents work by forming hydrogen bonds with cellulose fibers, enhancing the strength of the paper when wet. Natural wet-strength agents are generally less effective than synthetic agents but are often used in the production of high-end paper products such as tea bags, coffee filters, and cigarette paper.

The process of adding wet-strength agents to paper products varies depending on the type of agent being used. Synthetic agents are typically added to the paper slurry during the papermaking process, while natural agents are often applied to the surface of the finished paper product.

## 3. Mechanisms of Action of Wet-Strength Agents

Depending on their chemical composition, wet-strength additives can act as protective, reinforcing, and swelling prevention agents of the fibers, by protecting already existing bonds and/or by forming new water-resistant bonds [10]. Typically, the ability of an additive to impart water resistance properties to paper is related to four properties: (i) polymeric nature, (ii) water solubility, (iii) cationic character, and (iv) reactivity [12]. While reactivity is crucial and refers to the additive tendency to self-crosslink and form a water-resistant coating on the fibers, the positive charges themselves do not contribute to wet strength but allow for the initial anchoring of the additive to the fibers of anionic cellulose [13]. Once the additive adsorbs onto the fibrous substrate, it modifies: (i) the physical-structural properties of the fibers, through the formation of new fiber-fiber covalent bonds (*reinforcement mechanism*); (ii) the chemical properties of the fibers, making their surface hydrophobic or super-hydrophobic (*protective mechanism*) [14].

The chemical reactions which take place upon addition of the resin are: (i) the cross-linking of the cellulose or hemicellulose through the formation of resin-fiber covalent bonds; (ii) the reinforcement of the fiber-fiber contacts by forming a chemical lattice of the resin molecules which do not necessarily react with the functional groups of the fibers [10].

In the reinforcement mechanism, the agent reacts with cellulose or hemicellulose, forming covalent bonds between the molecules and fibers. These linkages supplement and strengthen the natural hydrogen bonding in the dry sheet, adding to the overall strength of wet fibers. Since these bonds are covalent, they are not broken by water. It is likely that the reinforcement mechanism involves some level of wet-strength agent crosslinking.

In the protective mechanism, the agent is dispersed onto the fibers and undergoes self-crosslinking to form an insoluble network that surrounds and penetrates the fiber contacts. This network impedes fiber separation when the paper is wetted, thereby preserving some of the original dry strength.

The mechanism of action of wet-strength agents depends on the type of agent used. Synthetic wet-strength agents such as polyamideamine-epichlorohydrin work by forming covalent bonds with cellulose fibers in the paper, resulting in a strong network that resists the breakdown of the paper when it comes in contact with water. Polyacrylamide works by adsorbing onto cellulose fibers and forming hydrogen bonds, while polyethyleneimine works by forming ionic bonds with the cellulose fibers. Natural wet-strength agents such as starch and chitosan work by forming hydrogen bonds with the cellulose fibers, enhancing the wet strength of the paper.

## 4. Synthetic Wet-Strength Agents

The most commonly used synthetic wet-strength agents are polyamideamine-epichlorohydrin (PAE), melamine formaldehyde (MF), polyacrylamide (PAM), glyoxylated polyacrylamide (GPAM), polyethyleneimine (PEI), polyvinylamine (PVAm) and polycarboxylic acids. These synthetic wet-strength agents can be used alone or in combination to achieve the desired wet-strength properties of paper.

The history of wet-strength additives in papermaking dates back to 1930 when PEI was first used, although its mechanism of action was unclear. Later, resins based on formaldehyde were developed, which were more cost-effective and efficient. However, their usage was limited due to their performance in only acidic conditions and the associated toxicity of formaldehyde. In the 1960s, PAE resins were introduced, which were known for their excellent performance under neutral and alkaline conditions. 

For most cationic resins, wet treatment of the paper consists of introducing the additive into the fibrous suspension before the formation of the fibrous mat. These resins are adsorbed by the fibers through electrostatic interactions that occur between the positively charged groups of the resin and the negative charges (carboxyl groups) of the lignocellulosic fibers. As the paper sheet dries, the polymer crosslinks under heating and a three-dimensional network is formed which gives the paper its moisture resistance. Depending on the synthetic product used, it is possible to obtain a permanent resistance to humidity, i.e., relatively unaffected by the increase in the time of contact of the paper with water, or a temporary resistance to humidity which decreases until it disappears as time increases contact of paper with water.

Many synthetic wet-strength additives are used at levels below 1% (*w*/*w*) based on dry fiber weight. Although wet-strength resins are added to impart wet strength, they also indirectly contribute to increasing the mechanical strength of papers in dry conditions.

### 4.1. Polyamideamine-Epichlorohydrin

**Figure 2:**
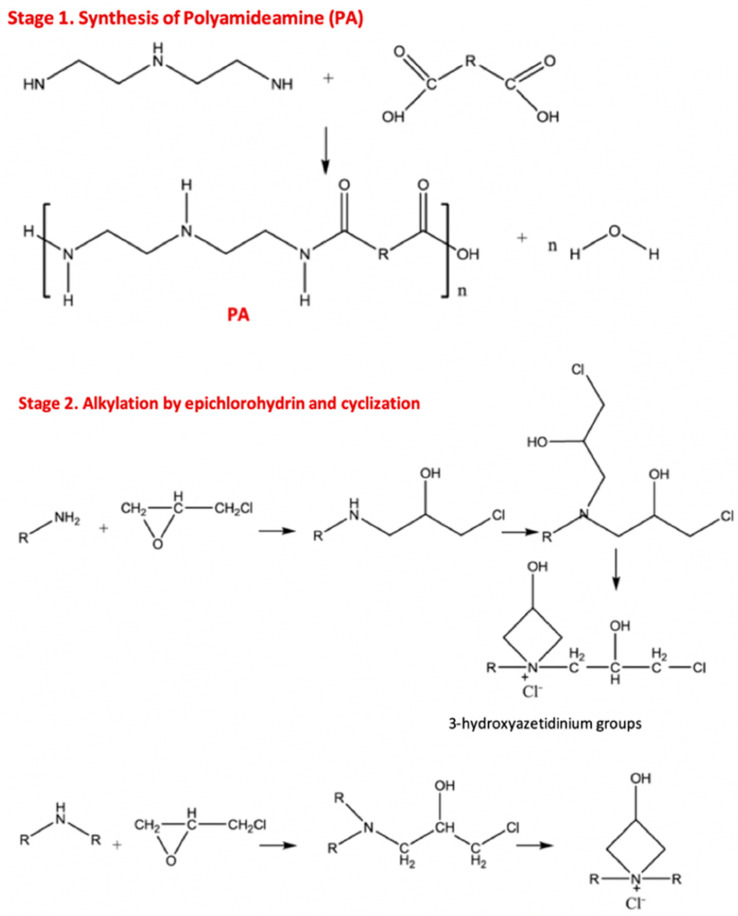
Scheme of polymerization of PA and its functionalization with epichlorohydrin to give PAE. The cationic azetidinium group is essential for additive adsorption on the cellulose fibers and for the crosslinking of PAE.

Due to its impressive wet-strength properties, good retention, and affordability, PAE is the primary synthetic wet-strength agent used today, despite having certain limitations that will be discussed later. This cationic resin is compatible with alkaline cellulose pulps and accounts for 90% of the wet-strength market [12]. PAE is synthesized through the polycondensation of adipic acid and diethylenetriamine to form a polyamideamine (PA), which is then functionalized with epichlorohydrin (Figure 2).

At the industrial scale, the polymerization and functionalization stages are carried out in consecutive steps. While most of the amino groups of the PA precursor are secondary, a small percentage (<5%) of primary amines and terminal carboxyl groups are also present. Epichlorohydrin reacts with these primary and secondary amino groups to create secondary and tertiary aminochlorohydrin groups, respectively. At neutral pH and temperatures above room temperature (60 °C), the tertiary aminochlorohydrin undergoes cyclization to form 3-hydroxyazetidinium groups (Figure 1). These stretched rings give the resin both reactivity and cationic charge.

**Figure 3:**
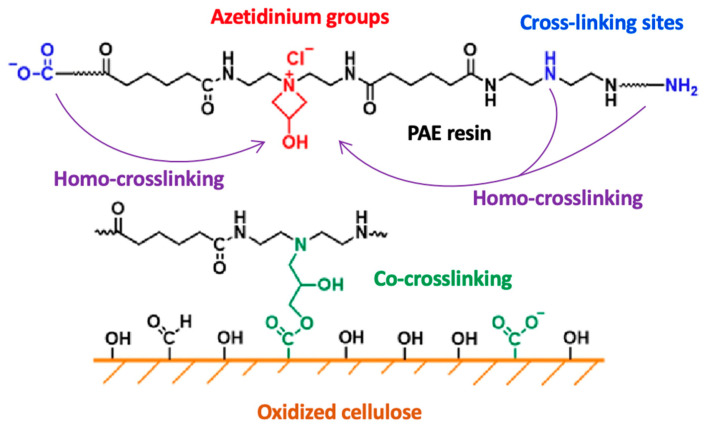
Reactive sites of PAE for co-crosslinking with oxidized cellulose (covalent bond between PAE azetidinium groups and cellulose carboxylic groups) and for homo-crosslinking (covalent bond between PAE free primary/secondary amines and azetidinium groups). Adapted from [15].

PAE resin provides resistance to wet essentially through two mechanisms: (i) resin/fiber co-crosslinking, which involves direct covalent bonding between cellulose fibers through a resin molecule, and (ii) resin-resin homo-crosslinking, which involves crosslinking of the resin with itself without forming covalent bonds with cellulose (Figure 3).

PAE azetidinium groups react with the carboxylic groups of cellulose for resin grafting onto cellulose fibers, while the free primary and secondary amino groups after functionalization with epichlorohydrin enable PAE auto-crosslinking, resulting in further mechanical strength and durability of the paper when wet. It is crucial that the functionalization phase of the PA resin with epichlorohydrin involves only a certain number of primary and secondary amino groups to remain free for optimal wet-strength properties.

PAE resin has good retention properties, which means that it remains in the paper fibers and does not become washed away during the papermaking process. This is important for maintaining the desired wet-strength properties of the paper.

**Figure 4:**
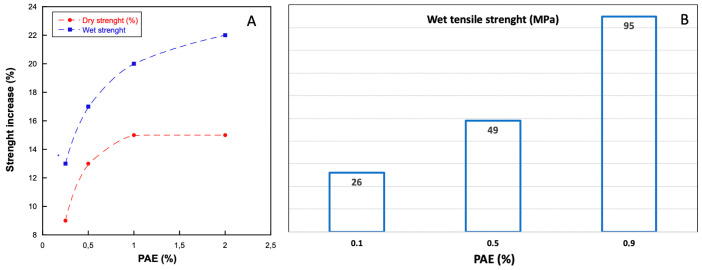
Effect of PAE dosage (%) on (**A**) the increase in wet and dry strength of softwood bleached Kraft pulp and (**B**) the increase in wet tensile strength of TEMPO-oxidized cellulose nanofibril.

In general, the increase in paper wet and dry strength depends on PAE dosage (Figure 4). The strength of the softwood bleached kraft pulp shows a noteworthy enhancement with a dosage of up to 1% PAE. At this dosage level, a remarkable improvement of 20% in wet strength and 15% in dry strength can be observed. However, beyond this dosage, the increase in wet strength becomes lower, while the dry strength remains unchanged [12].

TEMPO-oxidized cellulose nanofibril (TOCNs) films cross-linked with different dosages of PAE were also investigated [16]. The PAE/TOCNs film high dry tensile strength and Young’s modulus of approximately 135 MPa and 11 GPa, respectively. In addition, a high wet strength of 95 MPa was achieved for a 0.9% PAE content due to the cross-linked structure.

A drawback in the use of PAE is the potential presence of halogenated organic compounds (AOX), including epichlorohydrin, 1,3-dichloropropanol (1,3-DCP), and 3-monochloropropan-1,2-diol (3-MCP), even if recent technologies may drastically reduce the content of these compounds. For instance, the use of cellulose nanofibers in combination with PAE may reduce the wet-strength polymer quantity for development of more sustainable paper [17].

### 4.2. Melamine Formaldehyde Resin

**Figure 5:**
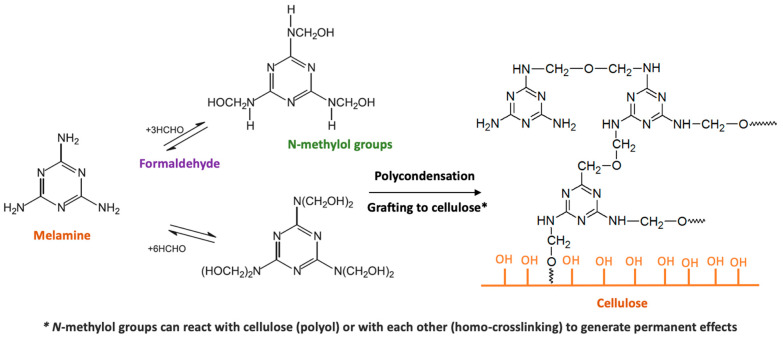
Schematic representation of polycondensation of formaldehyde and melamine and subsequent resin homo- and co-crosslinking with cellulose.

MF resin is also widely used in synthetic polymers as a wet-strength agent in the papermaking industry. It is prepared by the polycondensation of melamine with formaldehyde in the presence of an acid catalyst. The resulting polymer is then reacted with an acid or amine-functionalized epoxide to impart cationic charge to the resin (Figure 5).

The reaction mechanism of MF resin involves the formation of covalent bonds between the resin and cellulose fibers, as well as cross-linking between the resin molecules themselves. This results in improved fiber-fiber bonding, which enhances the strength and durability of the paper in wet conditions. One of the advantages of MF resin is it high degree of cross-linking, which results in a strong and durable network of covalent bonds that resists breakdown in wet conditions. Additionally, MF resin has good compatibility with both acidic and alkaline pulps, which makes it a versatile wet-strength agent for a wide range of paper grades.

MF resin also has good retention properties and is relatively low cost compared to other wet-strength agents. One of the main drawbacks of MF resin is its potential for formaldehyde release, which can pose health risks to workers in the papermaking industry. In response to these concerns, many paper manufacturers have switched to using alternative wet-strength agents that are formaldehyde-free. One approach is to partially replace formaldehyde with other aldehydes, such as glyoxal or glutaraldehyde, which have lower toxicity [18,19]. The reaction between dialdehydes and cellulose is catalyzed by metal or ammonium salts, among which the most used include aluminum, magnesium, and zinc salts. While glyoxal can increase the wet strength of paper, it may also lead to decreased flexibility, as evidenced by reduced stretch and folding endurance. On the other hand, glutaraldehyde treatment, particularly when a metal salt catalyst is used, can impart excellent wet strength to paper without sacrificing its folding endurance. Moreover, as the curing temperature during the glutaraldehyde treatment process increases, the wet strength of the paper can be further enhanced [15]. Another approach is to modify the MF resin with non-toxic, water-soluble polymers, such as polyvinyl alcohol (PVA) [20], which can reduce formaldehyde release [21]. PVA has also been used in combination with glyoxal or glutaraldehyde and was reported to increase the wet strength of the treated paper proportionally to its amount and molecular weight [22]. This indicates that the interaction between glutaraldehyde and PVA facilitates the formation of inter-fiber crosslinking, resulting in an improvement in wet strength while maintaining the flexibility of the paper [22].

### 4.3. Polyacrylamide and Glyoxylated Polyacrylamide

**Figure 6:**
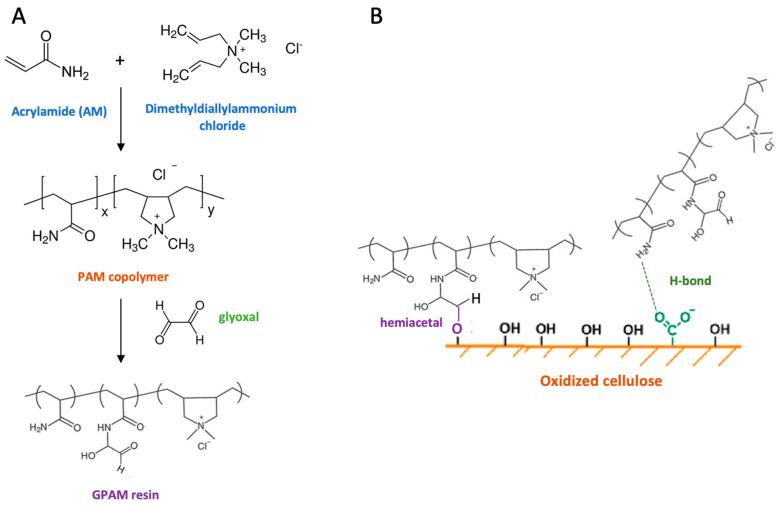
GPAM resin formation per reaction between a PAM cationic copolymer and glyoxal (**A**); GPAM H-bond and covalent interactions with cellulose (**B**).

PAM and GPAM are two commonly used synthetic polymers in the papermaking industry as wet-strength agents [23]. PAM is a water-soluble linear polymer synthesized from acrylamide monomers and is widely used as a flocculant, dispersant, and binder. GPAM is a modified form of PAM that contains glyoxal functional groups and has improved wet-strength properties. The new generation of GPAMs are obtained by a cross-linking reaction between different acrylamides and glyoxal. The most widely applied polymer is the copolymer obtained from dimethyldiallylammonium chloride (DMDAAC) and acrylamide (AM) [24], which has the characteristics of a cationic copolymer (Figure 6).

GPAM is synthesized by modifying PAM with glyoxal functional groups. The aldehyde glyoxal groups on GPAM react with the OH groups of cellulose fibers to form hemiacetals, which improve the wet strength of the paper.

The use of PAM and GPAM as wet-strength agents has several advantages over other synthetic polymers. Firstly, they do not contain formaldehyde or other toxic chemicals and are water-soluble, so can be easily added to the papermaking process. Secondly, they are compatible with a wide range of papermaking chemicals and can be used in combination with other wet-strength agents. However, compared to PAE, GPAM provides paper with a temporary protection to wet. Indeed, while PAE wet-strength decay rate will only be 10–15% after a paper sample is subjected to 30-min soak test, GPAM can provide much faster rates of decay in the range of 40–60% decay rate within a 2-min paper soak time [25]. This can be an advantage in the paper recycling process. The performance of PAM and GPAM as wet-strength agents can be improved by optimizing their molecular weight as well as by optimizing polymer composition. Increasing the molecular weight of the polymer may increase the number of potential bonding sites with the cellulose fibers, which improves the wet strength of the paper, but aggregation of the agent on the paper sheet may occur, which negatively impact the paper’s wet strength [21]. By properly combining molecular weight and cross-linking density, GPAM may be able to enhance paper’s wet strength better than PAE (GPAM 0.3% wet tensile index increased ratio of 381% versus 281% of PAE 0.5%) [26].

### 4.4. Polyethyleneimine

Polyethyleneimine is a synthetic cationic polymer that has been used as a wet-strength agent for paper products since the 1930s. PEI is a water-soluble polymer with a high molecular weight and a high charge density, which makes it strongly adhesive to the negatively charged cellulose fibers.

**Figure 7:**
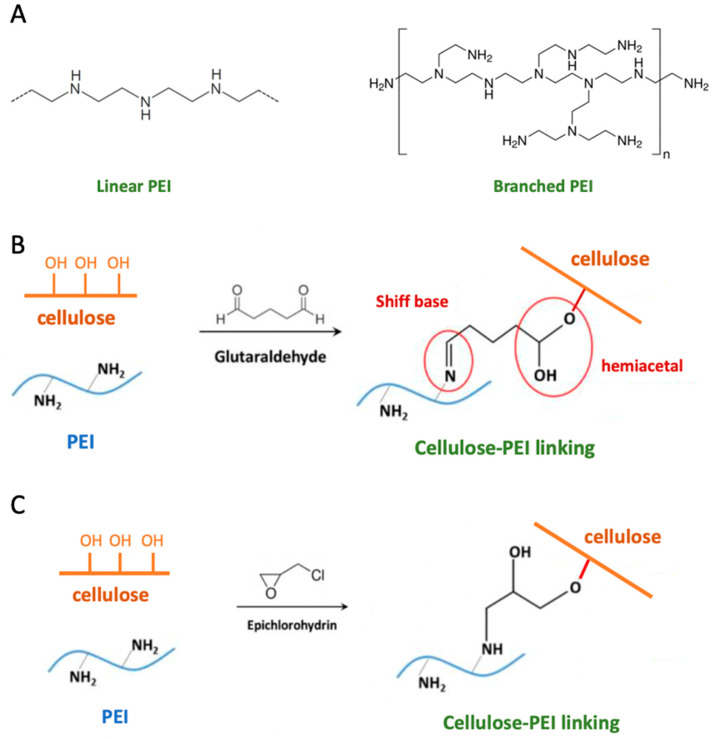
Linear and branched PEI (**A**); PEI-cellulose cross-linking by glutaraldehyde (**B**) or epichlorohydrin (**C**).

PEI is a linear or branched cationic polymer produced by the ring-opening polymerization of ethyleneimine (Figure 7). Branched PEI contains primary, secondary, and tertiary amines in the polymer backbone, with an amine density significantly higher when compared with most commercial cellulose-fiber coupling agents. That feature is essential for promoting covalent grafting of polymer chains on cellulose fibers and their cross-linking in reinforced composites.

To create a covalent bond between PEI and cellule fibers, different methods are available [27].

When starting from non-pre-functionalized cellulose, the use of cross-linkers is needed to obtain stable and durable composites. Glutaraldehyde (GAL) and epichlorohydrin are commonly used cross-linkers. GAL creates a cross-link between PEI and cellulose by forming a Schiff base and a hemiacetal (Figure 7).

This reaction can be conducted in a single step by stirring the solution in polar solvents, and can even be performed at room temperature. However, increasing the reaction temperature can result in a higher cellulose/PEI reaction, leading to a more stable product due to the increased amount of cellulosic polymer. The result is a three-dimensional network of cross-linked polymer chains and cellulose fibers that enhances the wet strength of the paper product.

When pre-functionalized cellulose is used, the direct cross-linking between the two building blocks is sometimes possible without additional cross-linkers. This is due to the presence of reactive functional groups on the cellulose fibers, such as carboxylic acid, amino or thiol groups, which can react with PEI. By adjusting the pH and temperature, it is possible to control the degree of cross-linking and the resulting properties of the composite.

One of the advantages of PEI as a wet-strength agent is its ability to improve the wet strength of paper products in both acidic and alkaline conditions. In addition, PEI has a low toxicity, is biodegradable, and is able to improve the ability of paper products to adsorb dyes. This is because the amino groups in PEI can react with dyes, resulting in a more uniform and vivid color. However, there are also some drawbacks associated with the use of PEI. One is the cost, as it is more expensive than other wet-strength agents such as PAE or GPAM. Additionally, the high cationic charge of PEI can interfere with the papermaking process, and PEI also has the potential for yellowing of the paper product over time, as it is sensitive to light and heat, leading to discoloration of the paper.

### 4.5. Polyvinylamine

**Figure 8:**
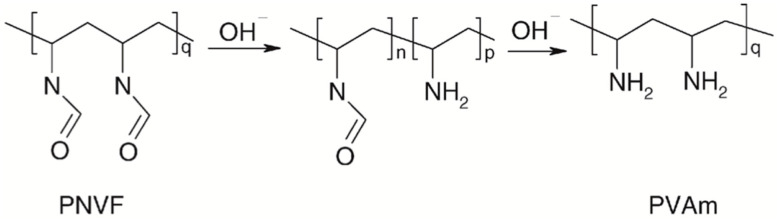
Structure of poly(N-vinylformamide)-polyvinylamine PNVF-PVAm copolymer obtained by basic hydrolysis of PNVF.

PVAm is a water-soluble polymer that confers wet-strength properties to paper products by adsorbing onto the paper fibers and creating a strong network that can resist the effects of water. The term PVAm refers to a series of poly(N-vinylformamide) (PNVF)-polyvylamine copolymers at different compositions obtained by a two-step procedure (Figure 8).

The first step is the synthesis of PNVF, which is a non-ionic water-soluble polymer. Then, PNVF is partially or fully hydrolyzed to give PVAm units bearing amine groups.

Weisgerber’s patent was likely the first to document the wet-strength properties of PVAm [28]. Lately, Pfohl [29] found that a 1 wt% PVAm solution in sulfite pulp (pine and beech mixture) at pH 7.5 yielded to a wet/dry strength ratio of 26%. Ten years later, Wang and Tanaka reported that using PVAm with an 11% hydrolysis level increased the wet/dry strength ratio of commercial hardwood bleached kraft pulp handsheets from approximately 5% to 30% [30]. Similarly, Pelton and Hong showed that newsprint treated with PVAm had a wet-strength value nearly 30% of the dry strength one. Notably, wet strength was unaffected by the degree of hydrolysis (56–100%) and remained constant for up to 1 h of soaking. The wet tensile strength was higher for paper treated at pH 10 compared to pH 3 or pH 7 [31].

The mechanism by which PVAm increases wet strength is not obvious since neither crosslinking nor grafting seem likely. DiFlavio and colleagues [32] reported that PVAm wet strengthening was approximately constant from pH 3 to 9, while it dropped at pH 3, where nearly every amine group on PVAm is charged (protonated), and at pH 9 where the PVAm is uncharged. That means that wet adhesion was independent of the extent of PVAm protonation (i.e., the charge content) over a broad range. Contrarily, the wet-strength adhesion was found to be related to the concentration of amino groups in the polymer backbone as well as to the cellulose oxidation degree. This supports the hypothesis that, besides the well-accepted electrostatic interaction between PVAm and cellulose fibers, the formation of covalent bonds between cellulose groups (acetal/hemi-acetals/aldehydes) and the PVAm amine is presumable to occur, as much as demonstrated for PEI.

### 4.6. Polycarboxylic Acids

**Figure 9:**
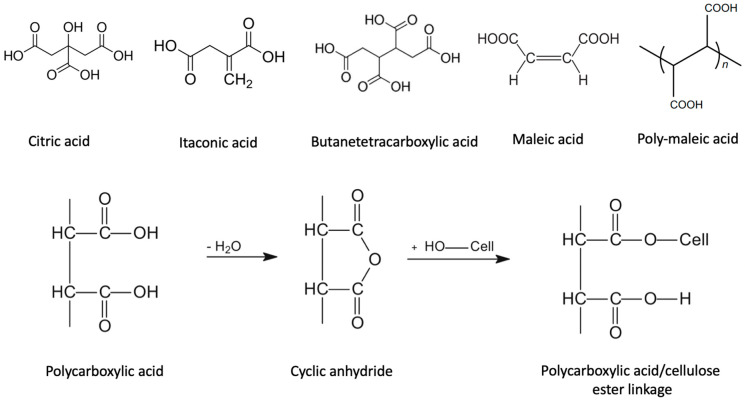
Main polycarboxylic acids investigated as crosslinkers or wet-strength agents for cellulose fibers and mechanism of action.

Poly(carboxylic acid)s have been used as crosslinking agents for cotton since the 1960s [33]. These compounds have carboxylic groups that can react with the hydroxyl groups of cotton cellulose, forming ester linkages and crosslinking the fibers. This crosslinking imparts improved properties to cotton fabrics, such as increased strength, durability, and wrinkle resistance. Among them, polycarboxylic acids such as 1,2,3,4-butanetetracarboxylic acids (BTCA) and citric acid are the most promising chemicals (Figure 9) [34].

Horie and Biermann reported in 1994 that bleached kraft handsheets treated with BTCA showed significantly improved wet strength [35]. The study found that treatment with BTCA improved the wet strength of the handsheets by a factor of 2–3, compared to untreated handsheets. The improvement in wet strength was attributed to the crosslinking of cellulose fibers by the ester bonds formed between BTCA and the hydroxyl groups on cellulose. Caulifield also studied the use of BTCA and citric acid for improving the dry and wet performance in this case of unbleached kraft board [36]. The study found that treatment with BTCA and citric acid improved the dry strength, wet strength, and folding endurance of the kraft board. Other interesting polycarboxylic acids are itaconic acid and maleic acid (Figure 9) that have been reported to be able to in situ polymerize and crosslink cotton fabric [37]. The expected cross-linking reaction between polycarboxylic acids and cellulose, occurring stepwise under heating, it is likely to begin with the transformation of the polycarboxylic acids into a cyclic anhydride, which is the real active species in the esterification with the hydroxyl group of cellulose (Figure 9) [38,39].

High-molecular-weight polycarboxylic acids, including polymaleic acid (PMA) and poly(methyl vinyl ether-co-maleic acid) (PMMA), have been used for wet-strength agents for paper products [40]. The effect of polymer molecular weight on wood pulp cellulose performance was investigated. The use of high-molecular-weight PMMA was shown to favor the formation of inter-fiber crosslinking, leading to an improvement in the dry strength and toughness of the treated paper. On the other hand, low-molecular-weight PMA tends to produce intra-fiber crosslinking, which can cause embrittlement of the fibers and reduce the flexibility of the treated paper [40].

## 5. Natural Wet-Strength Agents

Natural wet-strength agents for paper are derived from natural materials and in some applications can be used as an alternative to synthetic wet-strength agents. Some examples of natural wet-strength agents include starch, chitosan, cellulose nanofibrils (CNF), and soy protein. These natural wet-strength agents are biodegradable, renewable, and non-toxic, making them a more environmentally friendly alternative to synthetic wet-strength agents. However, they may be more expensive and have some limitations in terms of performance compared to synthetic wet-strength agents.

### 5.1. Starch

Starch is a natural polymer, made up of glucose units derived from various plant sources such as corn, wheat, and potatoes, which is commonly used as a binder in papermaking because of its ability to form hydrogen bonds with the cellulose fibers in paper. Thermoplastic starch is also used in packaging applications [41]. The formation of a network of hydrogen bonds with the cellulose fibers is believed to be the primary mechanism of action of starch as dry- and wet-strength agent [42]. Starch also has good film-forming properties, which help to coat and bind the fibers together, further improving the wet strength of the paper. The use of starch as a wet-strength agent is particularly effective in acidic paper-making conditions.

To achieve the high retention levels required in paper manufacture, starch has been functionalized in various ways to obtain cationic starch, oxidized starch, and esterified starch. Cationic starch is positively charged, available in different degrees of substitution, low (0.02–0.06) and high (≥0.07), obtained by the reaction of a primary hydroxyl etherification agent containing tertiary amines or quaternary ammonium groups, including glycidyltrimethylammonium chloride (GTAC) or 3-chloro-2-hydroxypropyltrimethylammonium chloride (CTAC) [43,44,45]. The adsorption of cationic starch on cellulose has been thoroughly investigated and was shown to enhance the strength of paper [46,47]. The electrostatic interaction of cationic starch and cellulose fiber can be affected by ionic strength, pH, and temperature [48]. More stable interactions can be achieved by adsorbing alternating layers of cationic and anionic starch where multilayers can be formed [49,50].

Cationic starch was found to be beneficial to the properties of PAE when added to pulp slurry, which led to a decreased PAE dosage for health concerns as food packaging materials [51].

Oxidized starch is instead produced by treating starch with an oxidizing agent such as sodium hypochlorite or hydrogen peroxide [52]. This process introduces carboxyl groups that make the starch more water-soluble and reactive [53]. In contrast, starch esterification with organic acids such as acetic or succinic acid makes it more hydrophobic and less water-soluble. Although starch is less efficient than synthetic wet-strength agents, in some applications, its use may be advantageous because it is a natural, renewable resource that is widely available and relatively inexpensive and does not affect paper recyclability.

### 5.2. Chitosan

Chitosan (CS) is a natural polycationic derived from chitin, a substance found in the shells of crustaceans. Chitosan is a biocompatible polymer which is also widely used in biomedical [49,50,51,52] and environmental applications [54,55,56,57,58,59,60]. Its use as a wet-strength agent was reported in papermaking in the 1990s [61]. Kraft paper coated with chitosan emulsion showed lower water vapor permeability rate (by ca 43%) and water absorption capacity (by ca 35%) compared to uncoated Kraft paper. The co-incorporation of palmitic acid further reduced the water vapor permeability rate and water absorption capacity of Kraft paper by 51% and 41%, respectively [62].

For uses in acidic pH, chitosan is not an ideal solution due to its tendency to partially dissolve in acidic environments. However, modifications can be made to chitosan to improve its chemical resistance using crosslinking agents such as glutaraldehyde, ethylene glycol diglycidyl ether, and epichlorohydrin [63]. The use of maleic anhydride-acylated chitosan, under various pretreatment and curing conditions, was investigated to improve the wet strength of handsheets. The results indicated that the highest wet-strength performance was achieved at a pretreatment pH of 6, and increasing the pretreatment temperature and polymer dose led to higher wet-strength index and an increased ratio of wet to dry strength. When compared to PAE resin, maleic anhydride-acylated chitosan showed slightly inferior results in terms of wet strength, with varied soaking durations. However, maleic anhydride-acylated chitosan still achieved about 80% of the wet strength of PAE, demonstrating its potential as a viable alternative to PAE for improving the wet strength of handsheets [63].

### 5.3. Cellulose Nanofibrils

In recent years, there has been an increased research interest surrounding cellulose nanofibril (CNF) as a natural nanocellulose material, owing to its biodegradability, barrier properties, chemical tunability, and exceptional mechanical properties [64]. Typically derived from plant fibers through enzymatic and/or chemical treatments, followed by physical treatments such as grinding or homogenization [65], CNF has proven to be a promising reinforcing agent for paper products [66,67].

In several studies, CNF was used as an anionic component of dual strengthening systems in combination with polymeric cationic wet-strength agents including PAE [68], cationic starch [69], and cationic polyacrylamide [70]. As far as PAE is concerned, due to environmental reasons there is an interest in decreasing the amount of PAE used in paper-making industry. Gardlund et al. [71] showed that the effect of PAE can be improved by adsorbing carboxymethyl cellulose (CMC), an anionic cellulose derivative, and PAE onto cellulose fibers. A further study investigated the use of CNF and PAE as agents to improve the wet and dry strength of paper [68]. Specifically, the adsorption behavior of CNF and PAE on cellulose model surfaces was analyzed using quartz crystal microbalance with dissipation (QCM-D) and atomic force microscopy (AFM). The study compared the layer structures and nano-aggregates formed by CNF and PAE onto cellulose fibers using different adding strategies. The results showed that when PAE was adsorbed first, a uniform and viscous layer of CNF could be adsorbed. However, when PAE and CNF were added as cationic aggregates, a non-uniform and more rigid layer was formed. The bilayer-adding strategy led to a significant increase in both the wet and dry tensile strength of paper even at low added amounts of PAE. On the other hand, the use of nano-aggregates did not lead to significant improvements in paper strength properties [68]. The utilization of a dual system consisting of PAE/CNF or CS/CNF resulted in significantly higher wet and dry tensile strengths of paper compared to using a single PAE or CS system. For instance, when the PAE (0.4%)/CNF (0.3%) dual system was used, the resulting paper exhibited 89% higher wet tensile strength than the paper using a single PAE (0.4%) system. Similarly, the CS (1.3%)/CNF (0.3%) dual treatment resulted in a 21% higher dry strength than the single CS (1.3%) system [69]. Recently, a cationic starch/CNF dual system was demonstrated to be superior to cationic polyacrylamide for improving the bagasse pulp and paper key properties [72]. This finding was related to the similarity of cationic starch and cellulosic fiber structure, as well as to the branched structure of cationic starch, in contrast to the linear cationic polyacrylamide.

### 5.4. Soy Protein

**Figure 10:**
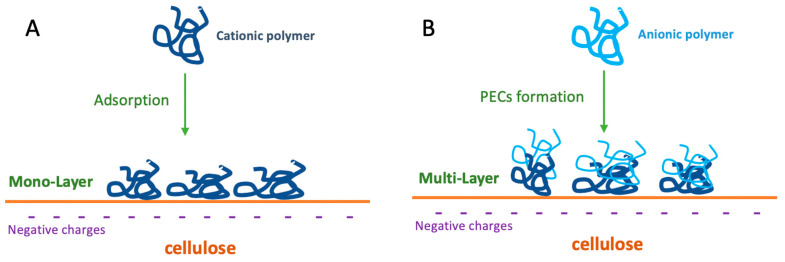
Strengthening of cellulose fibers by formation of polyelectrolyte complexes (PECs) onto the fiber surface by adsorption of cationic (**A**) and anionic (**B**) polymers.

Recently, soy protein has been proposed as an inexpensive, sustainable, and environmentally benign solution to enhancing papermaking performance, especially the dry strength [73,74]. As seen for CNF, also soy proteins have been used in combination with cationic polymeric strength agents, including cationic starch and chitosan [75]. Indeed, as described above, the use of polyelectrolyte complexes (PECs, Figure 10), obtained by a mixture of cationic and anionic polymers, have been shown to improve the mechanical properties of paper through increased electrostatic networking mechanisms among the PECs and fiber charged surfaces (Figure 10).

Both thermal-alkali degradation [76] and thermal acid treatment [77,78] could impart water-resistance properties to soy proteins due to the formation of water-resistant intermolecular chemical networks between proteins during treatment [79]. Li and Pelton [80] compared twenty proteins as potential paper wet-strengthening additives by measuring the peel force required to delaminate wet, regenerated cellulose films laminated with a thin protein layer. Results of the experiments varied greatly, indicating that the composition of the protein was a crucial factor in its effectiveness. The proteins with the highest levels of lysine and arginine exhibited the strongest adhesion, with additional contributions from hydroxyl and phenolic amino acid residues. Wet strength increased when laminates were cured at high temperatures (120 °C), suggesting that protein grafting to cellulose and protein cross-linking was important for good wet strength. Although none of the protein laminates was as strong as PVAm or PAE resins, the study suggests that increasing the content of cationic groups and optimizing heat-induced bond formation may lead to the development of a protein-based paper wet-strength resin in the future.

## 6. Techniques to Investigate the Effect of Wet-Strength Agents on the Physico-Chemical Properties of Paper Products

**Figure 11:**
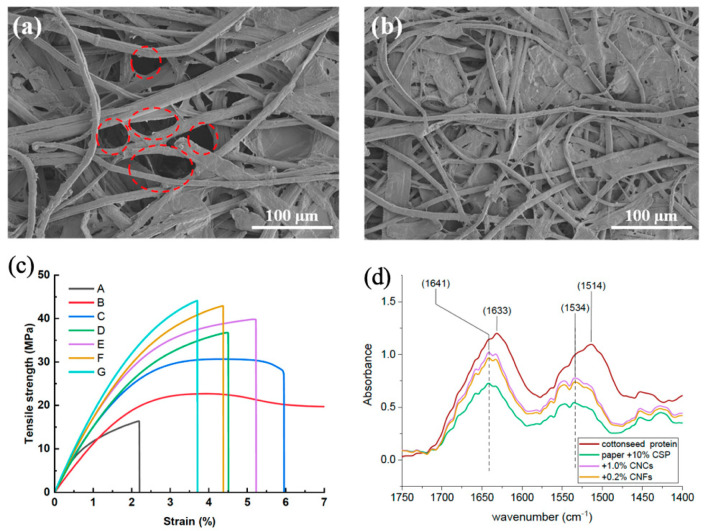
Examples of techniques used to investigate the effect of wet-strength agents on paper’s physico-chemical properties. (**a**,**b**) SEM micrographs of paper fibers (**a**) and paper fiber reinforcement by CMC-aGO (**b**) (red circles represent interfibrillar voids) (reproduced from [81]); (**c**) tensile strength–strain behavior of cellulose acetate butyrate (CAB)/acetone-treated cellulose nanofibers (A-CNF) with different amounts of the bio-derived crosslinker polyisocyanurate D376N. (A) A-CNF; (B) CAB500-5; (C) CAB/D376N (0 wt%); (D) A-CNF/D376N (3.9 wt%); (E) CAB/A-CNF/D376N (7.7 wt%); (F) CAB/A-CNF/D376N (14.3 wt%); (G) CAB/A-CNF/D376N (24.5 wt%). (Reproduced from [82]); (**d**) FTIR spectra of cottonseed protein (CSP) isolate and paper samples treated with CSP or CSF and nanocellulose (CNC), expansion of the cellulosic region of the amide bonds (1750 cm^−1^ to 1400 cm^−1^) (reproduced from [83]).

Different techniques can be used for investigating the performance of a specific wet-strength agent, including Fourier transform infrared spectroscopy (FTIR), X-ray photoelectron spectroscopy (XPS), scanning electron microscopy (SEM), tensile strength testing (INSTRON), dynamic mechanical analysis (DMA), and wet-peel and thermogravimetric analysis (TGA) (Figure 11).

### 6.1. Fourier Transform Infrared Spectroscopy

FTIR can be used to analyze the chemical bonds and functional groups present in the wet-strength agent and their interactions with the paper fibers. Obokata and Isogai investigated the mechanism of the wet-strength development of cellulose sheets prepared with PAE resin by FTIR. Specifically, PAE-containing cellulose sheets were first degraded by cellulase and FT-IR analysis was performed on cellulase-treated residues to investigate whether or not the covalent bond formation between azetidinium groups of PAE and carboxyl groups of cellulose fibers occurred. The influence of heating at 110 °C on covalent bond formation and paper wet-strength improvement was also studied [84]. In the FT-IR spectra of the cellulase-treated residues of both heated and unheated PAE-containing paper sheets, absorption bands were observed at 1735 cm^−1^ due to ester C=O groups and at 1550 cm^−1^ due to amide II N-H groups. In contrast, the FT-IR spectrum of the cellulase-treated residue of the blank sheets showed almost no absorption bands of this type. The heating process resulted in a clear increase in the amount of ester bonds present in the samples.

### 6.2. X-ray Photoelectron Spectroscopy

XPS is a surface analysis technique that uses X-rays to ionize the surface of a material and measure the energy of the electrons emitted from it. By analyzing the energy and intensity of the emitted electrons, XPS can provide information about the elemental composition, chemical bonding, and electronic states of the surface layer of a material. XPS can be used to study a wide range of materials, including metals, semiconductors, polymers, ceramics, and composites [85,86]. Chen and Tanaka applied XPS to investigate the surfaces of handsheets containing polydiallyl-dimethylammonium chloride (PDADMAC) and anionic polyacrylamide (A-PAM) as polymeric wet-strength additives, suggesting as this technique appears promising as a tool to analyze paper surfaces treated with small amounts of polymer additives [87]. XPS provided valuable insights into the covalent bonding between the strengthening agent and the fiber surface. This was achieved by analyzing the Nls chemical shifts observed in handsheets containing PDADMAC and PDADMAC + A-PAM. The use of XPS enabled a clear understanding of the chemical interactions between the strengthening agent and the fiber surface, shedding light on the bonding mechanisms involved [87]. Similarly, XPS was used to investigate CNF/carboxymethyl cellulose (CMC) composite film’s surface treated by glycidyl trimethyl ammonium chloride (GTMA) [88]. The reaction of GTMA with the hydroxyl groups of CMC by etherification was suggested by the XPS data.

### 6.3. Scanning Electron Microscopy

SEM can be used to examine the surface morphology and structure of paper fibers treated with the wet-strength agent, as well as the distribution in and penetration into the fibers of the wet-strength agent. The surface morphology of fibers after treatment with polymeric wet-strength agent can be significantly altered, as shown by observations of paper treated with either a cationic block waterborne polyurethane [89] or by mono- or di-methylomelamine (DMM) resin [90].

Specifically, SEM observation of untreated fibers of filter paper evidenced how they are interwoven by physical entanglement with the presence of many pores between fibers. In contrast, a dense film was observed on the same fibers treated with a cationic block waterborne [89].

Regenerated cellulose fibers treated by choline chloride and glycerol ionic liquid showed a more compact structure and fewer pores on the fiber surface, along with well-preserved cellulose frameworks compared to the untreated regenerated fibers. In addition, the tensile strength was improved from 54.43 MPa to 139.62 MPa after introducing the ionic liquid [91].

### 6.4. Tensile-Strength Testing and Wet Peeling

This involves testing the tensile strength of paper samples before and after being wet with water to determine the strength retention properties of the wet-strength agent [92,93]. Standard procedures are available to determine the wet tensile strength of paper or board. The ISO 3781:2011 (Paper and board—Determination of tensile strength after immersion in water) [94] and ASTM D 829-97 (Standard Test Methods for Wet Tensile Breaking Strength of Paper and Paper Products) [95] methods specify the apparatus and the conditions to be used for the determination of the wet tensile strength of paper after its immersion in water for a specified period. In addition, how to express the results in terms of wet tensile strength (*WTS*) and wet-strength retention (*WSR*) is reported. Wet-strength (*WS*) is routinely expressed as the ratio of wet to dry tensile strength (*DTS*) at break, while the wet strength retention is calculated as follows [96]:

$$WSR\;\left(\%\right)=\frac{WTS}{DTS}\times 100$$

The Instron machine can also be used for performing the so-called wet peeling test [97]. In this test, two wet and regenerated cellulose membranes are placed on top of each other with a thin layer of wet-strength resin in between. The stack is then pressed and dried before being rewetted. The resulting laminate serves as a physical model for studying wet fiber-fiber joints in paper. The strength of the wet laminate is finally measured by determining the force required to peel the two membranes apart at a 90-degree angle, also known as the peel delamination force. The wet-peel test conducted on TEMPO-oxidized cellulose membranes clearly demonstrated the impact of oxidation time on the wet-peel force. Notably, a substantial increase in wet-peel force was observed up to approximately 8 min of oxidation time. However, beyond this threshold, the wet-peel force exhibited minimal changes and remained largely unchanged [97].

### 6.5. Dynamic Mechanical Analysis

DMA can be used to study the mechanical properties of paper samples, such as modulus and damping, before and after being wetted with water. Wet-strength agent-treated paper may show a higher storage modulus (E’) than paper before impregnation, as shown for paper treated with poly (p-phenylene benzoisoxazole) (PBO) [98]. PBO not only conferred resistance to but also high thermal stability suitable for application in the aerospace industry.

DMA may also be a good method for examining whether an additive is molecularly distributed or making aggregates in paper and other composites. Indeed, the difference in the distributions of an agent within a fiber wall could deeply affect the strength development [99]. Mihara and Yamauchi showed the effect of polymer distribution on mechanical properties of paper sheets containing various amounts of cationic polyacrylamide dry-strength resin by DMA analysis performed in the 100–300 °C temperatures range in vibration mode at various frequencies [100]. DMA was a useful method for examining whether PAM was molecularly distributed or aggregating in the paper composites. The disappearance of PAM viscoelasticity was observed when PAM was distributed molecularly within the cellulose fiber wall. Conversely, when PAM was distributed around the fiber-to-fiber bonds or aggregated over the fiber surface, the viscoelasticity of the PAM phase became apparent.

### 6.6. Thermogravimetric Analysis

TGA can be used to investigate the thermal stability and decomposition properties of the wet-strength agent, as well as its compatibility with the paper fibers [101]. In general, a decrease in the additive compatibility with cellulose fibers has negative effects on paper performance. It is also possible to investigate cellulose fiber crosslinking by TGA. Generally, an increase in crosslinking density increases thermal stability of the material. However, in the case of polysaccharides, the perturbation of the H-bond network within the polymer chains by crosslinking may induce a decrease in material stability. This latter behavior was observed for chitosan [55] and also for hemicellulose/soy protein composite films cross-linked with citric acid [102]. Specifically, crosslinked hemicelluloses showed degradation temperatures lower than unmodified cellulose and related to the decrease in effectiveness of inter-molecular hydrogen bonds.

## 7. Challenges and Recent Advances

The development of effective paper wet-strength agents is an ongoing challenge in the paper industry. Synthetic wet-strength agents, in particular, are non-biodegradable and in some cases can pose a risk to human health and the environment. On the other hand, natural wet-strength agents are often not as efficient as the synthetic ones. Therefore, in recent years, there has been a growing demand for more sustainable and environmentally friendly wet-strength agents.

Most efforts in the development of wet-strength agents are focused on researching green precursors that are derived from bio-sources or waste materials. These precursors need to meet the main requirements for effective wet-strength agents, including solubility in water, the ability to bind to the surface of cellulose fibers, and reactivity to form a crosslinked and water-resistant coating.

Lignin, and its degradation products, is an interesting green precursor that can meet these requirements. Lignin is composed of a mixture of aromatic compounds, including hydrophobic polyphenols that provide structural support to plants. Although lignin is not water-soluble, it has a strong affinity for hydrophilic supports such as cellulose due to its ability to form hydrogen bonds [103]. Polyphenolic compounds, such as those found in lignin, are known to possess strong chelating and adhesive abilities [104], and also have interesting antioxidant and antimicrobial properties [105,106]. Thus, if used as wet-strength agents in papermaking, polyphenols could provide antimicrobial properties to the paper.

Another promising but still poorly investigated strategy towards sustainable wet-strength agents is the formation of supramolecular assemblies based on polyelectrolytes onto the cellulose fibers. Supramolecular assemblies, which are multi-component systems aggregated by noncovalent bonds, may exhibit properties not predictable from the features of the original constituents [107]. By rationally designing the colloidal entities, it is possible to tune the hierarchical self-assembly of the components [108]. By properly choosing the components, this approach could be used to create stable structures upon the surface of cellulose fibers to confer stability and dry/wet strength.

Another potentially promising strategy includes the use of enzyme-based approaches to modify cellulose fibers for improved wet strength [109]. Enzymatic modification appears to be a green and environmentally friendly process to change the properties of cellulose. Several enzymes, including laccases, esterases, lipases, and hexokinases, have been used for this purpose [110]. For instance, laccases have been extensively used for the modification of lignocellulosic materials, such as wood, sisal pulp, unbleached flax fibers, and softwood kraft pulp [111,112]. They are often employed with phenolic compounds to provide hydrophobicity, improved mechanical properties, and antioxidant and antibacterial properties.

## 8. Conclusions

Wet-strength agents play a critical role as additives in the paper industry due to their ability to enhance the mechanical properties of paper products when exposed to water. The availability of both synthetic and natural wet-strength agents in the market provides options for paper manufacturers, and their mechanisms of action vary depending on the specific type of agent used. In general, the additive effectiveness in providing water resistance to paper is influenced by four key properties: (i) polymeric nature, (ii) water solubility, (iii) cationic character, and (iv) reactivity. Reactivity plays a crucial role as it determines the additive’s ability to undergo self-crosslinking as well as to covalently bind to cellulose, forming a protective and water-resistant coating on the paper fibers. Positive charges themselves do not directly contribute to wet strength; however, they enable the initial attachment of the additive to the anionic cellulose fibers. By understanding and leveraging these properties, additives can effectively enhance the water resistance properties of paper and improve its overall durability and performance.

There is a need for further research and development to create wet-strength agents that are more efficient and cost-effective, addressing the growing demand for sustainable paper products. In recent years, the focus on environmental friendliness has gained significant importance in the papermaking industry. Many manufacturers are actively seeking wet-strength agents derived from renewable resources or those that have a minimal environmental impact. This shift in approach aims to reduce the ecological footprint associated with paper production and align with sustainable practices. By utilizing wet-strength agents derived from renewable resources, the industry can contribute to the preservation of natural ecosystems and reduce dependence on non-renewable resources.

Furthermore, the paper industry has the potential to leverage the opportunities presented by the bioeconomy in the coming years. The bioeconomy emphasizes the utilization of biomass, including forest resources, agricultural residues, and other organic waste materials, to produce a range of sustainable products. By embracing this concept, the pulp and paper sector can not only enhance its own sustainability, but also become a significant contributor to the transition towards a more sustainable and circular economy.

The exploration of alternative wet-strength agents that are both effective and environmentally friendly aligns with the broader goals of resource efficiency, waste reduction, and the development of a circular economy. The adoption of such agents would enable paper manufacturers to produce high-quality paper products while minimizing the environmental impact associated with traditional wet-strength agents. Additionally, this shift towards sustainable practices could enhance the paper industry’s reputation and appeal to environmentally conscious consumers who prioritize eco-friendly products.

In conclusion, the importance of wet-strength agents in the paper industry is clear. However, there is a need for ongoing research and innovation to develop more efficient and cost-effective agents. Embracing sustainable practices and the use of wet-strength agents derived from renewable resources can lead the industry towards a more environmentally friendly approach. By capitalizing on the opportunities presented by the bioeconomy, the pulp and paper sector can play a significant role in the transition to a sustainable and circular economy, ensuring a more sustainable future for the industry and the environment.

## Figures and Tables

**Table 1:** Chemical structures of main synthetic and natural wet strength.

Wet Strength Agents			
Synthetic Resin	Structure	Natural Resin	Structure
Polyamidemine-epichlorohydrin (PAE)	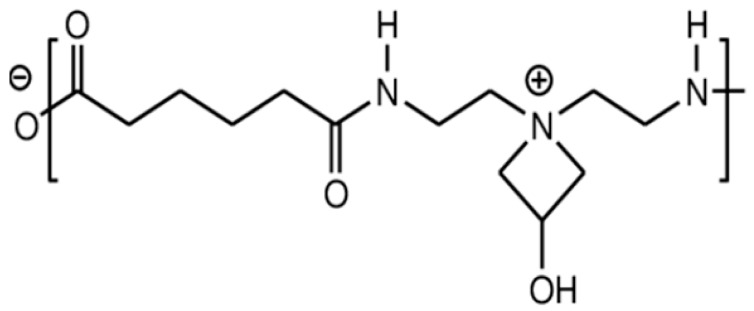	Starch	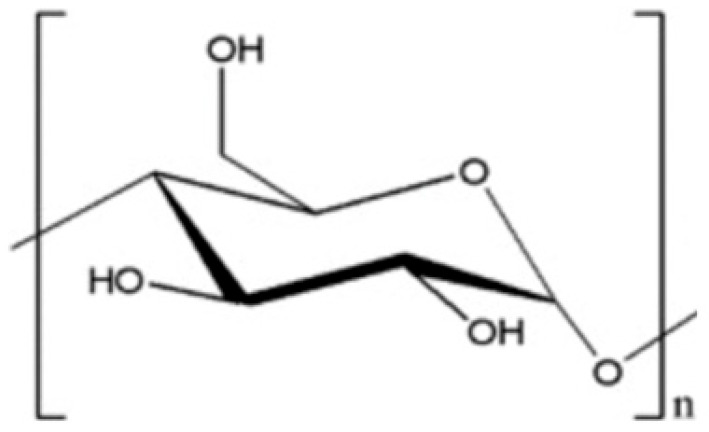
Melamine formaldehyde (MF)	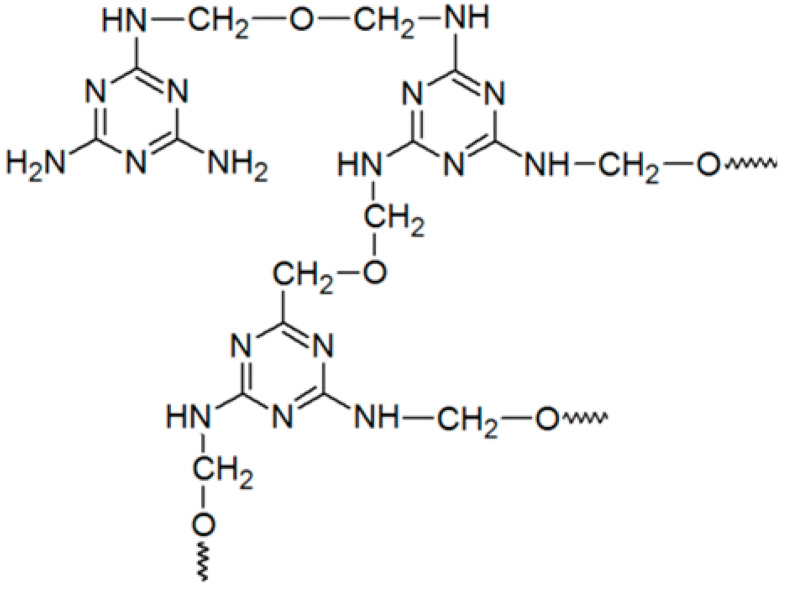	Cationic starch	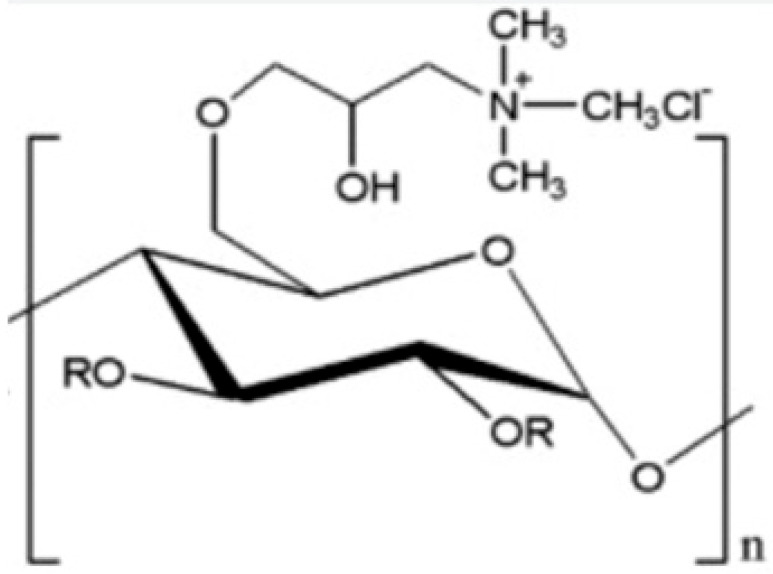
Polyacrylamide (PAM)	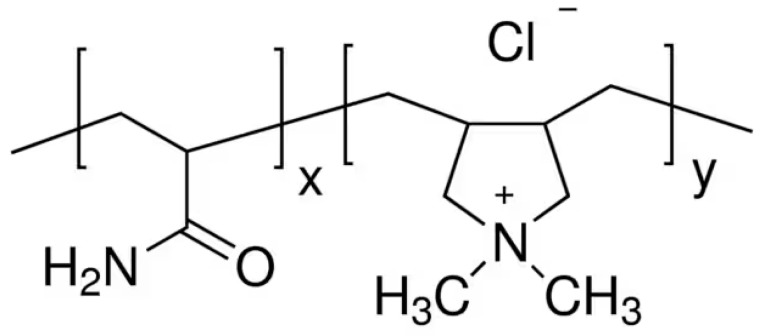	Chitosan	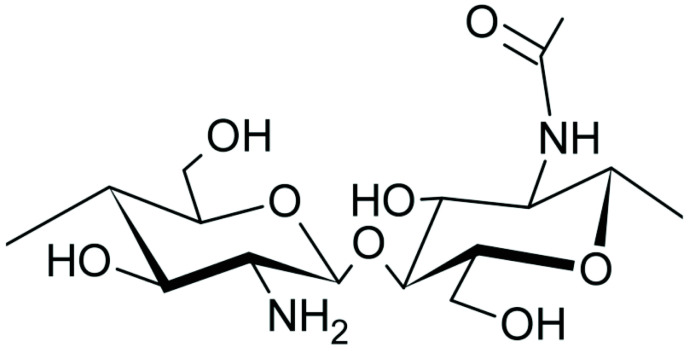
Glyoxylated polyacrylamide (GPAM)	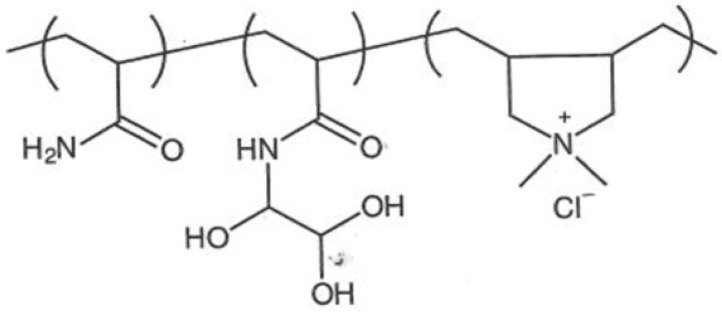	Cellulose nanofibrils (CNF)	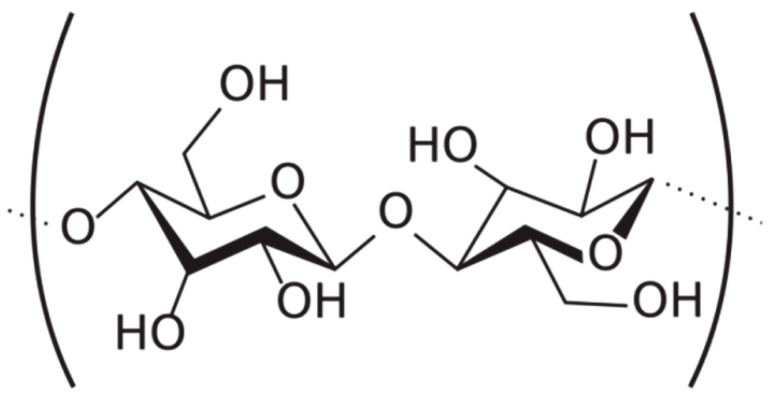
Polyethyleneimine (PEI)	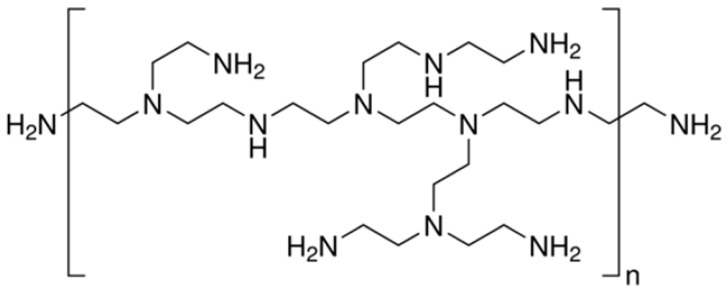	Soy protein	-
Polyvinylamine (PVAm)	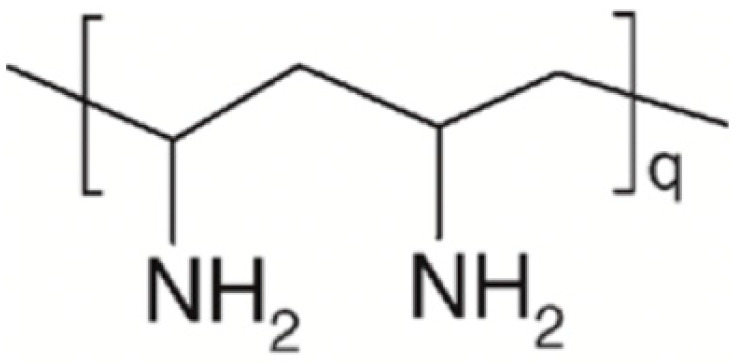	Lignin	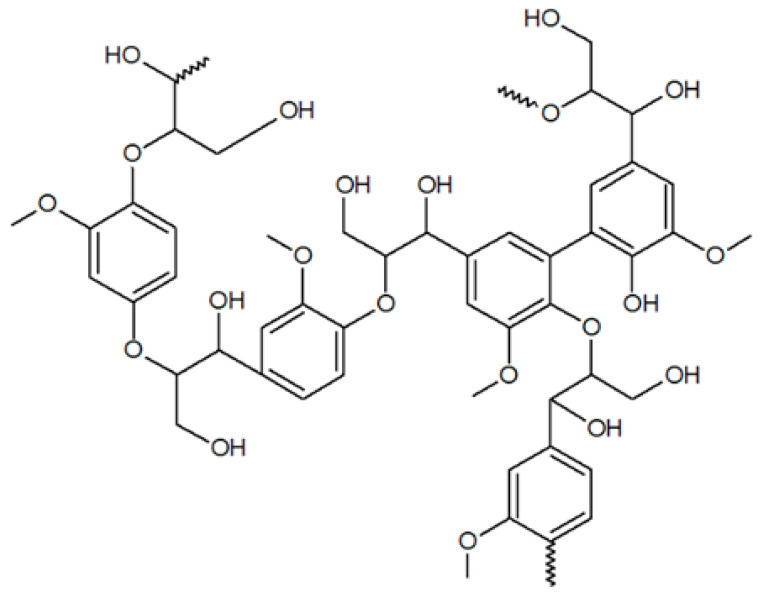

## Data Availability

Not applicable.
